# An Immersive Virtual Reality Simulation Program for Dementia Caregivers: A Technical Report and Pilot User Feedback

**DOI:** 10.7759/cureus.115934

**Published:** 2026-09-08

**Authors:** Sarah C Pistritto, Krystina M Clarke, Andrei Torres, Mary Chiu, Irmina Klicnik, Cole G Craven, Asmi Khanna, Nusrat Choudhury, Bill Kapralos, Amer M Burhan

**Affiliations:** 1 Faculty of Health Sciences, Ontario Tech University, Oshawa, CAN; 2 Faculty of Computer Science and Technology, Algoma University, Brampton, CAN; 3 Toronto Dementia Research Alliance, Ontario Shores Centre for Mental Health Sciences, Ontario Tech University, Whitby, CAN; 4 Research Department, Ontario Shores Centre for Mental Health Sciences, Whitby, CAN; 5 Cell and Systems Biology Department, University of Toronto, Toronto, CAN; 6 Medical Devices Research Centre, National Research Council Canada, Boucherville, CAN; 7 Medical Education and Simulation Department, maxSIMhealth Group, Ontario Tech University, Oshawa, CAN; 8 Psychiatry Department, Temerty Faculty of Medicine, University of Toronto, Toronto, CAN

**Keywords:** acceptability, caregiving, dementia, feasibility, simulation, tolerability, virtual reality

## Abstract

Caregivers often navigate complex behavioral and communication challenges with limited opportunities for guided practice, making scalable, skills-based training a persistent need in dementia care. This technical report summarizes the 12-month iterative development of VR SIM Carers, an immersive virtual reality (VR) simulation program for caregivers and care partners of people living with dementia. The program delivers psychoeducational content through interactive, first‑person VR scenarios that allow caregivers to practice communication and decision-making in realistic home‑based care situations. The report outlines the user‑centered, iterative development process, key technical and design decisions, and refinements informed by multi‑cohort user testing. As a secondary component of the technical report, pilot user testing with 24 caregivers provided preliminary information on feasibility, acceptability, usability, and tolerability, and helped identify considerations for deploying and refining VR-based caregiver training in clinical and community settings.

## Introduction

Simulation-based education has become a cornerstone of contemporary healthcare professions' education and training, providing structured opportunities to rehearse complex skills and decision-making in safe, controlled environments [1]. Within this paradigm, virtual reality (VR) offers an immersive, interactive modality where users can maneuver three-dimensional (3D) multimedia sensory environments in real time [2]. Recent evidence shows that VR application in healthcare professions education is emerging as a promising modality that can improve participants' knowledge and skills [2], yet its application to caregiver education in dementia remains limited. Existing dementia care education for unpaid family caregivers and care partners is often delivered through didactic formats or low-fidelity digital resources, which, although beneficial, may be difficult to access, insufficiently tailored to daily challenges, and constrained in their capacity to support practice of communication and problem-solving skills in emotionally charged situations [3,4]. This has resulted in a pressing need for evidence-informed, scalable, and accessible VR tools in this space.

The Reitman Centre for Alzheimer's Support and Training at Mount Sinai Hospital (Toronto, CA) developed the CARERS program (Coaching, Advocacy, Respite, Education, Relationship, Simulation) that provides a structured, evidence-based framework that targets practical caregiving skills, communication strategies, problem-solving, and emotional coping, and has demonstrated improvements in caregiver competence, coping, burden, and mental well-being through the use of problem-solving techniques and simulation-based skills training [5,6]. Building on these principles to address these described limitations, the VR-SIM Carers program was developed as an evidence-informed, scalable VR simulation program designed to deliver and strengthen psychoeducational support for unpaid family caregivers and care partners of people living with dementia (PLwD) in community settings [7]. Anchored in principles of simulation-based education, the program aims to provide realistic, hands-on practice with common caregiving challenges. It focuses on enhancing caregiver self-efficacy, adaptive coping, relational communication, and the management of behavioral and psychological symptoms of dementia (BPSD) through guided, experiential learning, while enabling evaluation of the feasibility, acceptability, and tolerability of immersive VR as a modality for dementia caregiver education.

This technical paper describes the end-to-end development of VR-SIM Carers, a VR simulation program designed to deliver psychoeducational content to family caregivers and care partners of PLwD. The primary purpose of this report is to document the design rationale, technical specifications, development process, and iterative refinements that shaped the prototype. Pilot user testing with caregivers was included as a secondary component to inform refinement and provide preliminary information on feasibility, acceptability, usability, and tolerability. These findings are presented to contextualize the development process and guide future evaluation, rather than to make claims regarding efficacy or clinical effectiveness. While we describe development, implementation, and preliminary user testing to support future evaluation, we do not intend to make any claims of efficacy.

## Technical report

System design

Hardware and Software Used

The VR-SIM Carers program was developed in Unreal Engine 5 (by Epic Games [8]) and deployed using Meta Quest headsets. Testing was conducted across multiple devices (e.g., Meta Quest Pro, Quest 3, and Quest 2 headsets) to ensure compatibility and performance across the various Meta Quest headsets. Facial motion was captured using the Live Link Face app (by Epic Games [9]) on an iOS device, which recorded facial expressions and voice acting for character animation, which was later applied to MetaHumans (a framework by Epic Games for creating high-fidelity digital humans - see Figure 1), enhancing realism and emotional engagement within scenarios.

**Figure 1:**
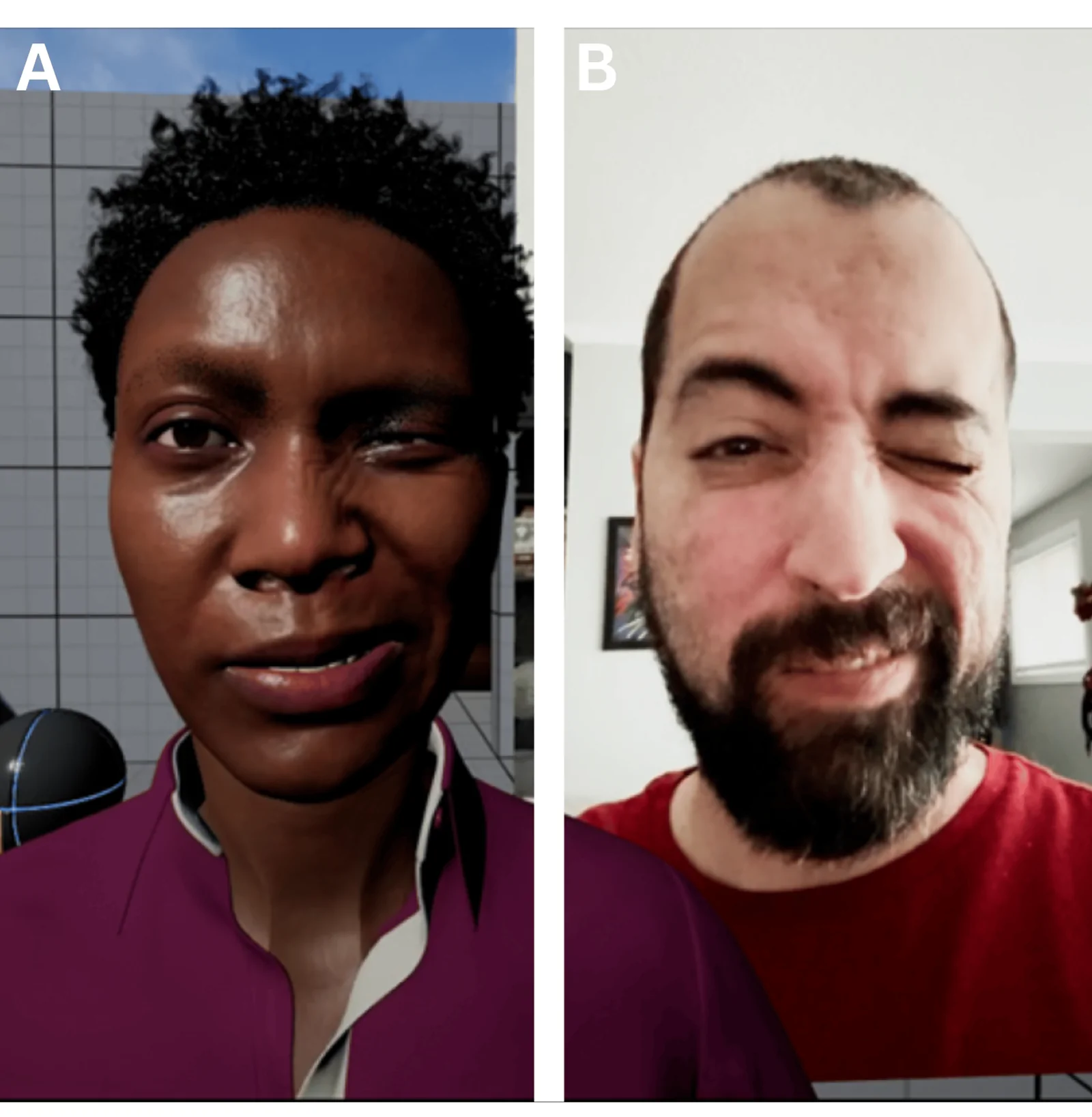
Example of facial expression mapped to a MetaHuman character. (A) A high-fidelity MetaHuman character rendered in Unreal Engine. (B) A researcher performing a specific facial expression, which is mapped in real time to the MetaHuman character using performance capture technology.

Design Principles

Development followed user-centered and participatory design principles [10], with strong emphasis on accessibility. Early prototypes explored multiple locomotion options, such as free teleportation, shift movement, and smooth locomotion. Based on feedback from the steering committee, free locomotion was removed to reduce cognitive and physical burden on users. The final design uses a teleportation-based navigation system, whereby users are automatically moved to locations based on their dialogue choices. This approach minimizes motion discomfort and simplifies interaction. Subtitles were incorporated into the scenarios with solid backgrounds for readability, and all dialogue is accompanied by audio narration to support diverse user needs.

Environment Development

Each environment was developed using a staged approach, beginning with greyboxing to establish layout and spatial flow, followed by incremental addition of props, lighting, and visual detail. Environments evolved over time as project needs changed. Earlier builds supported full locomotion, whereas later versions relied entirely upon teleportation, allowing environments to be scaled more efficiently while reducing collision and navigation complexity. 

Audio and Voice

Ambient sound effects (e.g., background television audio) were incorporated to reflect everyday caregiving contexts. Early development also explored text-to-speech AI-generated voice systems (using ElevenLabs [11]); however, final implementations prioritized clarity and consistency of narration and character dialogue. Therefore, we have adopted speech-to-speech (via ElevenLabs), in which we provided the narration for each character, conveying the proper tone, cadence, and emotion that each scene requires, and converted it into a different-sounding voice to match the virtual characters. That allowed us to solve the issue of having synthesized voices and narration that sounded monotonous and unrealistic. No AI service was used to generate scenario/clinical content or participants' content.

Content and Scenario Design

Psychoeducational content (including modules and scripts) was developed by a content creation team and then refined and adapted for immersive VR delivery. A key early challenge involved translating traditionally written, film-style scripts into interactive, first-person role-playing experiences suitable for VR. This required extensive iteration to support branching dialogue and user choice. To support this process, node-based mapping tools (e.g., MindMeister [12]) were initially used to visualize dialogue structures, as their format closely aligned with the visual scripting system of the Narrative Toolset, the Unreal Engine plugin selected for this project (version 0.3.9). These maps facilitated the development of non-linear narrative pathways and interactive decision points relying on various events or conditions for the user within each scenario.

Three scenarios were developed for this initial prototype. Each used linear logic with branching, meaning that there was one path, with branches that eventually led back to the main path. There were no parallel paths. The three scenarios were as follows: 

Managing apathy - addressing engagement, function, and quality of life: Users sit beside an avatar representing a mother living with dementia. The objective is to engage her in a meaningful conversation. Answers can be selected from a menu, and depending on which response is selected, the mother character will respond differently. The scenario also features an avatar representing Aiden, a figure that will discuss what happened in the scenario. Both Aiden and the mother will provide feedback on the interaction.

Refusal of care - navigating barriers to safe and effective treatment: Users adopt the role of the primary caregiver to an avatar representing their father living alone with dementia. This father has had his cognitive abilities decline over time. When the end user visits the father, they will notice smoke coming from a pan on the stove. End users will select responses and options from a menu to make the environment safe and de-escalate the situation. Similar to the "Managing Apathy" scenario, Aiden is also present and will provide feedback to the end user, as will the father avatar.

Crisis intervention (smoking pan) - rapid decision-making to prevent harm: The user will sit with an avatar representing their father living with dementia. The objective is to get the father to get ready to go out for his usual morning walk; however, he is wearing dirty clothes. Again, end users will have the ability to select responses from a menu and be provided with feedback from both Aiden and the father avatar.

Scenarios were chosen with input from members of the research team who had experience working with people living with dementia and their caregivers [13]. Figure 2 shows a screenshot of the crisis intervention (smoking pan) scenario from two points of view. Additional screenshots of scenarios have been published elsewhere [7]. Together, these scenarios enable the prototype to address both routine care and safety considerations, providing a balanced framework to support caregiver judgment, communication, and intervention strategies in real-world contexts.

**Figure 2:**
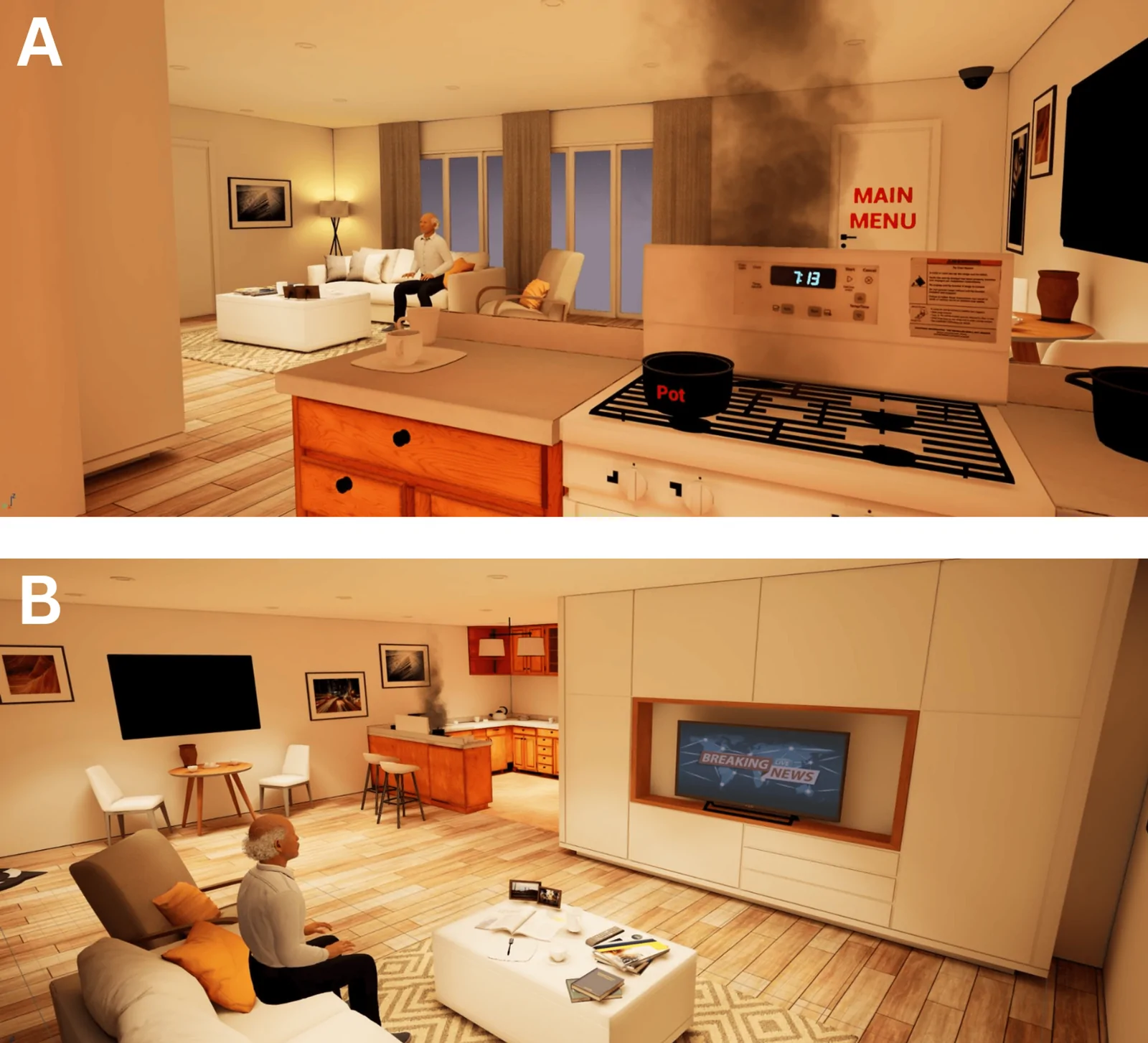
Two points of view of the crisis intervention (smoking pan) scenario. (A) Kitchen viewpoint showing the stove hazard. (B) Living room viewpoint showing the overall apartment layout and the seated avatar.

A summary of these decisions is provided in Table 1. 

**Table 1:** Key technical decisions summary. This table presents a concise breakdown of the final design choices implemented in the simulation, alongside the alternative options explored and the underlying justifications for each selection.

Decision	Options Explored	Final Choice	Rationale
Locomotion	Free teleportation, shift, smooth locomotion	Auto-teleportation to dialogue choice	Reduced cognitive load and motion sickness
Voice Generation	Text-to-speech (TTS)	Speech-to-speech (S2S)	Monotone TTS lacked emotional nuance; S2S preserved tone and cadence
Dialogue	Film-style (linear) script	Node-based (non-linear) branching (MindMeister)	Supported non-linear user agency and decision points
Environment	Full detail up front	Greyboxing, add incremental detail	Enabled rapid iteration and layout validation before committing art resources
Subtitles	Optional overlay	Always-on, solid background	Accessibility; accommodates hearing difficulties and noisy environments

Iterative development and refinement

An iterative development approach, over a period of approximately 12 months, was central to the project workflow (Table 2). For each scenario, multiple development approaches were tested, evaluated, and refined through continuous feedback and technical experimentation. Environments were frequently revised, typically producing two to four versions per scenario to ensure alignment with narrative requirements and technical constraints. As development progressed, substantial changes were made to both interaction mechanics and visual design, reflecting insights gained from prototyping, testing, and feedback from the steering committee. This process enabled progressive refinement of usability, performance, accessibility, and narrative coherence across scenarios. Initial participants in Cohort 1 (n = 5) and Cohort 2 (n = 5) reported usability and immersion challenges with the VR-SIM Carers program during their check-in calls, including navigation difficulties, inconsistent audio, reduced avatar realism, and headset discomfort (see Sections "Cohort 1 Issues and Fixes" and "Cohort 2 Issues and Fixes"). These issues were addressed through iterative refinements, and no similar concerns were reported in subsequent Cohort 3 (n = 4), Cohort 4 (n = 5), and Cohort 5 (n = 4), suggesting effective resolution and data saturation for user experience themes.

**Table 2:** Iterative development approach of VR-SIM Carers scenarios, co-design with caregivers and clinicians.

Months	Development
1-3	Clinical content development: Three scenario scripts developed by a team of subject matter experts (i.e. clinicians experienced with dementia care)
4-12	VR Scenario development: Building VR environment and characters; Voice recording and speech-to-speech conversion; Facial expression recording; Demonstration to / feedback from caregiver knowledge users; and Iterative changes based on feedback from caregiver knowledge users

Cohort 1 Issues and Fixes

Interface and navigation: (a) Third menu option in Scenario 3 (S3) was too low to select reliably - repositioned; (b) exit door in S3 did not function - fixed; and (c) no resume/pause button for reflection periods - added.

Audio: (a) Static and background noises reported in S3 - audio cleaned; and (b) inconsistent volume across characters - normalized.

Character and avatar: (a) Dr. Aiden perceived as stiff, "wooden-like", "jarring", with a distracting holographic circle - revised animation and visual framing; (b) characters not blinking - added blink animation; and (c) S2 dad's hands hovering over his lap, which broke immersion - pose corrected.

Environment: (a) Environment was described as resembling "hotel rooms" - adjusted props and furnishing for domestic realism; (b) lag between interaction and response - optimized event triggers; and (c) spelling errors throughout "gangerous" for "dangerous", missing K in "knife and fork" - corrected.

Side effects: (a) 2 of 5 described the headset as "heavy"; 2 of 5 reported eye strain - break guidance added to onboarding.

Cohort 2 Issues and Fixes

Interface and navigation: (a) Exit button in S2 was difficult to activate - hit target was enlarged; (b) speech bubbles covered the field of view and obscured the NPC dad - repositioned and resized; (c) dialogue options cut off in second half of S2 and S3 - text container was expanded; and (d) Dr. Aiden's menu appeared in front of her while seated, blocking view - UI placement adjusted.

Audio: (a) S3 had static/background noise for 2 participants, TV audio too loud - further audio pass completed; and (b) audio gradually got louder in S2 - volume curve corrected.

Character and avatar: (a) Dr. Aiden's holographic appearance was distracting, especially in S1 where she appeared more transparent than in S2 and S3 - visual consistency normalized; (b) less emotional expression across avatars; inconsistent eye contact and lip sync between scenarios - animation pass applied; (c) father (Vincent) had visible hair glitch (full hair when leaning forward, balding when leaning back) - mesh fixed; and (d) mother character perceived as androgynous with distracting neck vein detail - character model revised.

Feedback and content: (a) Recaps and feedback felt repetitive, especially in S2 and S3 - consolidated to a summary with optimal responses; (b) users requested the ability to skip dialogue on static menu - skip functionality and persistent menu added; (c) S3 referenced a white shirt and hat which were not present in the environment - props added or dialogue corrected; and (d) headset crashed when removed mid-session with no recovery - resume-from-checkpoint functionality added.

By the final cohorts, participants reported no new usability concerns, indicating that the platform had reached functional stability and that data saturation was achieved for user experience themes.

Pilot user testing

Method

The VR-SIM Carers prototype was piloted with 24 unpaid family caregivers and care partners of PLwD to inform early stages of refinement and to assess usability. The VR-SIM uses brief headset-based dementia caregiving scenarios with dialogue choices, feedback, and branching pathways to support reflection and skill practice. Participant recruitment occurred through Baycrest, Ontario Shores Centre for Mental Health Sciences, London Health Sciences Centre, and CareFirst, leveraging existing clinical and community networks serving family caregivers and care partners of PLwD.

After a brief onboarding process (including headset setup, Simulator Sickness Questionnaire (SSQ) [14] screening, and completion of three introductory simulations), participants used the program independently at home on a provided Meta Quest 2 or Meta Quest 3 headset. Participants selected scenarios through a main menu and interacted with the VR environment using a variety of dialogue interfaces; each choice activated predefined events and subsequent branches, enabling personalized narrative progression. Session duration varied depending on the exploration of dialogue paths. Typical use ranged from 10 to 15 minutes.

The primary outcomes (feasibility, usability, acceptability, and tolerability) were monitored through brief check-ins ranging from 5-20 minutes, and post-intervention assessments, including the Game User Experience Satisfaction Scale (GUESS-18) [15], the Igroup Presence Questionnaire (IPQ) [16], and the SSQ to assess user experience, presence, and any adverse effects lasted between 30-60 mins. Table 3 outlines the check-in phone call questions. 

**Table 3:** Check-in phone call sample questions.

Area	Sample question	Optional prompt/probe
Technical issues	Have you encountered any technical issues with the VR headset?	Is the headset charged? Are you having any issues connecting to the internet or starting the scenarios?
Headset care	Have you had any questions about how to care for or store the headset?	Is it being turned off and placed back in the box after use? Have you had any difficulty cleaning it?
Comfort/safety	Have you experienced any unbearable discomfort while wearing the VR headset?	What did you do when you experienced the discomfort? Were you sitting down? Did you take a break? Did you return to the scenario afterward?
Scenario completion	Which VR scenarios have you been able to complete so far?	Were there any scenarios you had difficulty starting or completing?
Virtual characters	How have you found the virtual characters, such as Aiden, to interact with?	Did you find them friendly or comfortable to interact with?
Audio	How was the audio during the scenarios?	Was it clear and easy to understand?
Visual environment	How was the visual environment in the VR scenarios?	Could you see the environment and characters clearly?
Learning	What have you learned so far about dementia caregiving from the scenarios?	Has anything stood out to you or changed how you think about dementia caregiving?
Progress reminder	Have you had a chance to start any of the VR scenarios?	If not, is there anything preventing you from getting started?
Assessment tools	Have you completed the assessment tools?	If not, would you like us to resend the link or schedule a call to walk through them with you?
General concerns	Do you have any other concerns or questions about the study or the VR headset?	Is there anything we can help you troubleshoot or clarify?
Need for follow-up	Is there anything you would like us to follow up with you about?	-

Feasibility: It was defined as the ability of participants to complete onboarding and independently use the VR-SIM Carers program in a home setting. Feasibility was considered met if ≥80% of participants were able to complete the VR-SIM Carers program without researcher assistance after onboarding.

Usability: It was assessed using the GUESS-18 [9], a validated 18-item self-reported instrument designed to evaluate subjective experience with the interactive system. Items are rated on a 7-point Likert scale (1 = strongly disagree to 7 = strongly agree), with higher scores indicating a more positive user experience. Mean scores are calculated for each subscale and for the overall scale, resulting in values ranging from 1 to 7. Scores closer to 7 indicate higher user satisfaction and more favorable experiential outcomes, whereas lower scores reflect reduced usability and engagement. Usability was also assessed qualitatively, alongside feedback captured during our check-in phone calls.

Acceptability was defined as participants' perceived satisfaction, engagement, and willingness to use the VR-SIM Carers program again. Acceptability was considered met if intervention adherence was 75% or more, completing at least two of three scenarios and reviewing virtual clinician feedback during the at-home loan period [7]. Acceptability was assessed by the IPQ [10], a 7-point Likert scale instrument including both Likert-type and bipolar formats, with appropriate reverse scoring applied where required. The questionnaire produces three primary subscales (spatial presence, involvement, and experienced realism), as well as a single-item general presence rating. Subscale scores are computed as mean values, with higher scores (range: 1-7) indicating a stronger sense of presence, greater attentional engagement, and higher perceived environmental realism. There are no established clinical cut-offs; therefore, scores are interpreted comparatively across conditions, with higher values reflecting stronger immersive experience. Acceptability was also informed by user feedback from qualitative data received from participants.

Tolerability was defined as the absence of clinically meaningful simulator sickness or adverse effects during or after use. Tolerability was assessed using the SSQ scale, a 16-item instrument that measures symptoms commonly associated with VR. Participants rated symptom severity on a 4-point scale (0 = none to 3 = severe). Weighted scoring procedures were used to compute three subscale scores (nausea, oculomotor, disorientation) and a total severity score. Higher scores indicate greater simulator sickness severity and reduced tolerability of the VR experience. The SSQ total score is commonly interpreted as follows: minimal symptoms (0-5), mild symptoms (5-10), moderate symptoms (10-15), and pronounced symptoms (>15), although no formal clinical thresholds exist.

Tolerability was considered conditionally acceptable if 16-30% (5-9/30) of participants reported moderate symptoms that were transient and remediable without intervention discontinuation [7]. Tolerability was considered unacceptable, warranting cessation or major redesign, if more than 30% (>9/30) of participants reported moderate or severe symptoms or if any serious adverse event attributable to the intervention occurred [7].

The pilot was conducted under multi-site REB approval (Ontario Shores Centre for Mental Health Sciences Joint Research Ethics Board (JREB 22-031-B), with secondary approval from the National Research Council Research Ethics Board (NRC-REB 2023-23). All participants provided written informed consent. 

Participants 

In total, 24 individuals consented to participate and completed all of the VR SIM scenarios, but not all completed the remaining four study processes (see Figure 3). Twenty-three provided baseline demographics, 22 completed quantitative questionnaires, 23 participants completed qualitative semi-structured post-intervention interviews, and 21 participants completed one-month follow-up responses.

**Figure 3:**
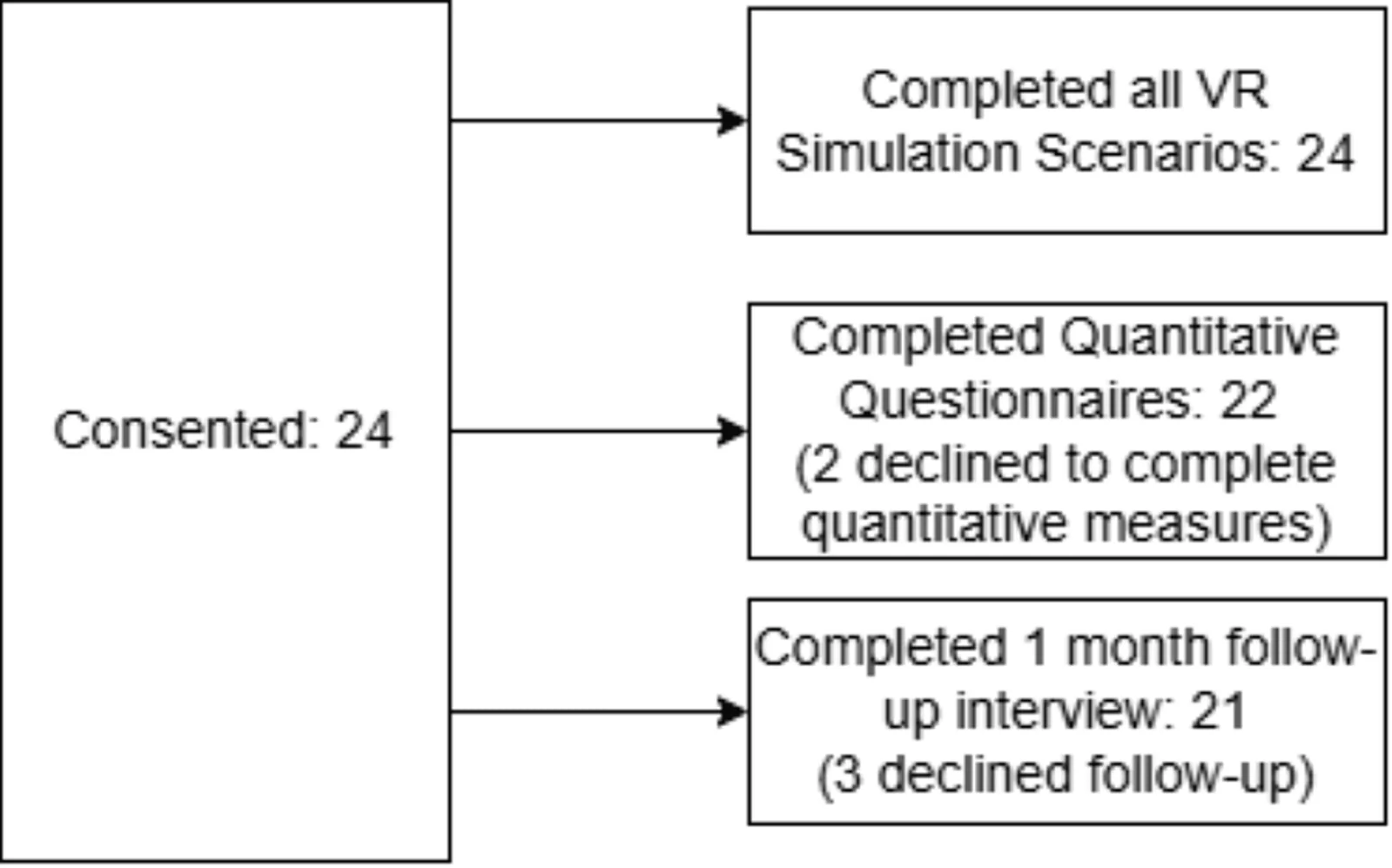
Participant flow diagram. VR: virtual reality

Baseline demographic data were available for 23 caregivers. Participants had a mean age of 57.09 years (SD: 13.68; range: 25-77), representing caregivers across early, mid, and later adulthood. The majority identified as women (60.87%). The sample was 73.91% White and 34.78% East Asian, alongside representation from other racial groups. The response options were not mutually exclusive, allowing participants to identify all categories that best represented their culture.

Over half of participants (65.22%) described themselves as the primary caregiver, and most were adult children caring for a parent with dementia, followed by spouses. Care recipients resided across various settings, including cohabitation with the caregiver and long-term care or retirement residences.

Caregiving duration ranged from three months to 15 years, capturing individuals at multiple stages of the dementia trajectory. Notably, 56.52% of participants reported no prior formal dementia training, and none had previous exposure to VR-based caregiver education, highlighting both the heterogeneity of caregiving experience and the novelty of the VR-SIM Carers intervention within this cohort.

Quantitative Data Results 

All participants (100%) completed the onboarding and scenarios independently; therefore, the feasibility threshold of >80% was achieved.

SSQ results: A total of 24 participants completed the SSQ during orientation. The mean total SSQ score was 10.59 (SD = 13.67), indicating the presence of significant simulator-related symptoms across the sample. While the mean SSQ score (10.59) fell within the moderate symptom range, substantial variability was observed (see Table 4 and Table 5). Subscale analysis revealed differing symptom profiles. Nausea scores were low (M = 3.58, SD = 6.78), suggesting negligible nausea-related symptoms among participants. In contrast, oculomotor symptoms were higher (M = 11.05, SD = 15.32), indicating significant visual strain and fatigue associated with the virtual environment. Similarly, disorientation scores were elevated (M = 13.92, SD = 19.25), reflecting significant symptoms, such as dizziness and spatial instability. Notably, the relatively large standard deviations across subscales suggest substantial inter-individual variability in symptom experience. Simulator sickness was considered acceptable when participant SSQ total scores remained below 15, consistent with previous VR usability studies reporting low levels of simulator-related discomfort.

**Table 4:** Simulator Sickness Questionnaire (SSQ) scores and acceptability following VR exposure (n = 24).

SSQ Outcome	Mean (SD)	Median (IQR)	Range	95% CI
Total SSQ score	10.59 (13.67)	3.74 (18.7-0)	0-44.88	4.82-16.36
Nausea subscale	3.58 (6.78)	0 (9.54-0)	0-28.62	0.72-6.44
Oculomotor subscale	11.05 (15.32)	3.79 (17.06-0)	0-53.06	4.58-17.52
Disorientation subscale	13.92 (19.25)	6.96 (17.4-0)	0-69.6	5.79-22.05

**Table 5:** Participant distribution according to SSQ acceptability threshold. SSQ: Simulator Sickness Questionnaire

SSQ severity classification	Definition	Participants, n (%)
No symptoms	SSQ = 0	10 (41.67%)
Negligible simulator sickness	0 < SSQ < 5	3 (12.5%)
Minimal simulator sickness	5 ≤ SSQ < 10	2 (8.33%)
Significant simulator sickness	10 ≤ SSQ < 15	2 (8.33%)
Symptoms are a concern	15 ≤ SSQ ≤ 20	2 (8.33%)
Above acceptable threshold	SSQ > 20	5 (20.83%)

While most participants reported minimal discomfort, a subset exhibited higher levels of oculomotor strain and disorientation. Overall, these findings suggest that although the VR-SIM intervention was generally tolerable, specific domains of simulator sickness (Table 6), particularly visual strain and disorientation, may warrant further optimization in future iterations of the platform.

**Table 6:** Frequency and severity of simulator sickness symptoms following VR exposure (n = 24). VR: virtual reality

Symptom	Participants reporting symptom, n (%)	Severity observed
Eyestrain	7 (29.2%)	Mild-moderate
Difficulty focusing	6 (25.0%)	Mild-moderate
Blurred vision	6 (25.0%)	Mild-moderate
General discomfort	5 (20.8%)	Mild
Fullness of head	3 (12.5%)	Mild
Headache	2 (8.3%)	Mild
Sweating	2 (8.3%)	Mild
Fatigue	1 (4.2%)	Mild
Increased salivation	1 (4.2%)	Mild
Difficulty concentrating	1 (4.2%)	Mild
Dizziness (eyes open)	1 (4.2%)	Mild
Vertigo	1 (4.2%)	Mild
Stomach awareness	1 (4.2%)	Mild
Nausea	0 (0%)	None
Dizziness (eyes closed)	0 (0%)	None
Burping	0 (0%)	None

IPQ results: IPQ data were available for 22 participants. Descriptive statistics are presented in Table 7. On the −3 to +3 scoring scale, general presence had a mean score of 0.76 (SD = 2.14) and a median of 1.00 (IQR = 3.50). Spatial presence had a mean of 0.49 (SD = 2.01) and a median of 1.00 (IQR = 3.00), while involvement had a mean of 0.10 (SD = 1.97) and a median of 0.00 (IQR = 3.00). Experienced realism had the lowest central tendency, with a mean of −0.83 (SD = 1.91) and a median of −1.00 (IQR = 4.00). REAL1 was excluded from the experienced realism subscale calculation.

**Table 7:** Mean scores (M) and standard deviation (SD) for I-Group Presence Questionnaire (IPQ) subscales. Note: Scores are reported on a seven-point scale coded from -3 to +3, with higher scores indicating greater presence. Scores reflect average responses within each IPQ subscale. REAL1 was excluded from the realism subscale calculation. GP1: General Presence item 1; SP1-SP5: Spatial Presence items 1-5; INV1-INV4: Involvement items 1-4; REAL1: Realism item 1

Subscale	Items	Mean (SD)	Median (IQR)	95% CI	Interpretation
General Presence	GP1	0.76 (2.14)	1 (3.5)	−0.19 to 1.71	Low-moderate presence
Spatial Presence	SP1–SP5	0.49 (2.01)	1 (3)	−0.40 to 1.38	Low spatial presence
Involvement	INV1–INV4	0.10 (1.97)	0 (3)	−0.77 to 0.97	Low involvement
Experienced Realism	REAL2–REAL4 (REAL1 excluded)	-0.83 (1.91)	-1 (4)	−1.68 to 0.02	Very low realism

GUESS-18 results: GUESS-18 data were available for 21 participants. Descriptive statistics for the eight available GUESS-18 subscales are presented in Table 8. Usability/playability demonstrated the highest mean score (M = 5.71, SD = 1.02), followed by personal gratification (M = 5.12, SD = 1.52) and narrative engagement (M = 5.10, SD = 1.52). Enjoyment had a mean score of 4.79 (SD = 1.59), while audio aesthetics, visual aesthetics, and play engrossment had mean scores of 4.50 (SD = 1.62), 4.40 (SD = 1.66), and 4.36 (SD = 1.66), respectively. Creative freedom had the lowest mean score (M = 4.10, SD = 1.57; n = 20). Social connectivity could not be evaluated because items 17 and 18 were not available in the dataset. 

**Table 8:** Descriptive statistics for Game User Experience Satisfaction Scale (GUESS-18).

GUESS-18 subscale	n	Mean (SD)	Median (IQR)	Range	95% CI
Usability/playability	21	5.71 (1.02)	6.00 (5.00-6.50)	3.00-7.00	5.25-6.18
Narrative engagement	21	5.10 (1.52)	5.50 (4.50-6.00)	1.00-7.00	4.40-5.79
Play engrossment	21	4.36 (1.66)	4.00 (3.50-5.50)	1.50-7.00	3.60-5.11
Enjoyment	21	4.79 (1.59)	4.50 (3.50-6.00)	2.50-7.00	4.06-5.51
Creative freedom	20	4.10 (1.57)	4.00 (3.38-5.00)	1.00-7.00	3.37-4.83
Audio aesthetics	21	4.50 (1.62)	4.50 (4.00-6.00)	1.00-7.00	3.76-5.24
Personal gratification	21	5.12 (1.52)	5.50 (4.00-6.50)	2.00-7.00	4.43-5.81
Visual aesthetics	21	4.40 (1.66)	4.50 (4.00-6.00)	1.00-7.00	3.65-5.16

Quantitative data findings are summarized in Table 9.

**Table 9:** Quantitative data summary. GUESS-18: Game User Experience Satisfaction Scale; IPQ: Igroup Presence Questionnaire (IPQ); SSQ: Simulator Sickness Questionnaire

Measure	Key Finding
Simulator Sickness (SSQ)	Generally tolerable; subset reported visual strain and disorientation
Presence (IPQ)	Low-moderate; realism perceived as limited
User Experience (GUESS-18)	High usability and personal gratification; lower creative freedom

Qualitative Findings

One-month follow-up field note key lessons learned: Participants were asked four questions at one-month follow-up, with the question focus and purpose of exploration listed in Table 10, and responses summarized from field notes in Table 11. Due to one incomplete dataset and one loss to follow-up, n = 21 participants were included in this qualitative one-month follow-up analysis.

**Table 10:** Field note questions. PLwD: people living with dementia

Question Number(s)	Focus of Question(s)	Purpose of Exploration
1-3	Strategies learned through the program	To examine what strategies caregivers acquired
1-3	Impact on caregiver well-being and competence	To assess perceived changes in caregiver confidence, skills, and emotional well-being
1-3	Perceived behavioral or attitudinal changes in the PLwD	To explore observed changes in behaviors, mood, or interactions
4	Additional reflections or insights	To capture perspectives, experiences, or observations not addressed in earlier questions

**Table 11:** Thematic mapping of responses to the VR-SIM Carers one-month follow-up program feedback (n = 21). Note: These field notes were used descriptively to illustrate participant experiences and guide refinement rather than for formal content analysis. Therefore, theme frequencies were not calculated and the key lessons should not be interpreted as consensus findings. PLwD: people living with dementia; VR: virtual reality

P#	Key Strategies Learned	Impact on Caregiver	Observed Changes in PLwD	Additional Notes/Reflections
001	Validate, reassure, redirect	Less confrontational, mindful of reassurance	PLwD more dependent, better interaction	VR headset cumbersome; prefers YouTube role-play
002	Pause before responding, listen actively	Felt more competent	Less angry outbursts	Enjoyed trying scenarios
003	Calm voice, eye contact, simple tasks, 3R’s, inclusion in activities	More understanding, patient, forgiving self	Father more receptive, relaxed	Learned acceptance of daily variability
004	Effective listening, eye contact	Increased confidence	More responsive, patient	Appreciated awareness gained
005	Caring approach, meeting needs	Lowered stress, more confident	Minimal changes yet	—
006	Anticipate needs, cognizant of thought deterioration	More competent, balanced personal/time needs	PLwD speech declining	Appreciated VR experience
007	Distraction, physical touch	Less frustrated, positive visits	Stronger eye contact, better engagement	Interested in more VR segments
009	Patience, change topics	Easier to appease	No change	-
010	Avoid open-ended questions, silence with physical contact	Easier, manageable	Calmed her	More assistance in public/erratic situations
011	Face PLWD, stay calm	Easier communication, calmer management	PLwD calmer	-
013	Perspective-taking, present-moment awareness	Increased empathy, resilience, patience	PLwD less agitated, easier bathing/changes	Transformative experience
015	Greet, eye contact, calm voice, breathe, respect, patience	Lowered stress, more competent	PLwD relaxed, cooperative	Scenarios “pop up” in real life
018	Patience, temper control	Both caregiver and PLWD calmer	Less shouting	-
019	Validate feelings, diversion	Still stressed, feel incompetent	Little change	Learned to pick battles, lower expectations
020	Anticipate behavioral responses, multi-modal communication, trial/error	More adaptable, understanding	Better task compliance sometimes	Desired more challenging VR scenarios
021	Calm down, pause before replying, seek support	Improved quality time	PLwD less argumentative, relaxed	VR scenarios intense but reinforced strategies
022	Calm voice, limit questions, break tasks	Lowered stress, improved relationship	Reduced PLwD anxiety, better cooperation	VR fostered empathy, reinforced self-care
023	Redirect, stay calm, patient	Less guilt, better equipped	Reduced agitation, better relationship	Realized common caregiver experiences
024	3R’s (Recognize, Reassure, Redirect)	Reduced stress, structured approach	PLwD calmer, cooperative	Learned to prioritize self-care
025	Reassure, redirect	Lowered stress, more at ease	PLWD compliant	Enjoyable and positive interactions
027	Awareness of apathy	Lowered stress, less internalization	No behavioral change	Found VR innovative, needs more practice

Responses provided an overview of how participants applied VR-SIM Carers strategies and their perceived psychosocial and relational outcomes. As a technical report, the scope of this evaluation is bound to the immediate, localized experiences of our specific deployment cohort, rather than broad, generalizable behavioral trends.

The VR-SIM Carers program revealed several key lessons (summarized in Table 11): (1) Communication: calm, patient, and simplified communication strategies improve engagement and reduce agitation in PLWD. (2) Emotional regulation: caregiver self-care and stress management directly affect caregiving effectiveness. (3) Behavioral impact: PLwD responsiveness can improve through reassurance, redirection, and structured interactions, though outcomes may vary. (4) VR-SIM efficacy: immersive VR scenarios support experiential learning, enhance empathy, and provide practical skills applicable to daily caregiving.

## Discussion

Limitations

As this is a technical report describing pilot refinement rather than an efficacy trial, the absence of a control or comparison group limits causal interpretation of participant-reported outcomes. Outcomes reported in this study relied on self-reported caregiver experiences, including perceived changes in caregiving strategies, stress levels, and interactions with PLwD, and were not independently verified through behavioral observation or clinical measures. Early technical issues related to audio quality, interface navigation, and avatar realism were reported by participants in the first cohorts. Although these issues were addressed during the iterative refinement process, they may have influenced initial user experiences. Continued technological improvements will further enhance immersion and usability. The use of text-to-speech voices may lack natural emotional tone and expressiveness, potentially reducing realism and emotional immersion in the VR scenario, which may have influenced participants' engagement and empathic responses. Finally, the present study primarily examined caregiver experiences rather than direct clinical outcomes for PLwD. Future research should explore whether VR-based caregiver training leads to measurable improvements in caregiver burden, patient behavioral symptoms, and overall quality of life.

Next steps

These next steps reflect both directions for our own future work and opportunities for others to build on and extend this work. Several of these steps are already underway as part of ongoing refinement of the program over the past two years, while remaining items represent planned priorities for future development. First, we will be expanding the scenario library beyond the initial three to cover a broader range of caregiving challenges, such as wandering and difficult conversations around driving, including scenarios that may require additional communication strategies. Planned improvements to the platform include increasing avatar realism through enhanced facial expressions, body language, and dynamic responses; exploring voice-interactive dialogue in place of menu-based selection; developing more meaningful choose-your-own-adventure branching; integrating facilitated discussion or live role-play alongside VR sessions; and adding a pause-and-resume function to support note-taking and reflection. Beyond improving the platform itself, longitudinal evaluation of behavioral change and outcomes for PLwD should be conducted to determine whether the learning gained through the iVLE can be sustained over time. Finally, evaluation of deployment feasibility across clinical and community training settings will also be needed. Taken together, these priorities will support the continued development, evaluation, and uptake of the platform across training contexts.

## Conclusions

Pilot user testing, included as a secondary component of the development process, provided preliminary information indicating that caregivers were able to complete the program independently and generally perceived the platform as usable, acceptable, and relevant to caregiving practice. Through iterative refinements across cohorts, early usability challenges were identified and addressed, and later participants reported fewer technical barriers, suggesting improved functional stability of the VR-SIM platform for caregiver training. Participants described learning practical communication strategies and reported perceived changes in confidence, emotional regulation, and interactions with PLwD; however, these self-reported findings should be interpreted as exploratory user feedback rather than evidence of program effectiveness. While this initial feedback is encouraging, the iterative build-test-feedback cycle described in this report was essential to achieving a usable prototype and should be considered an important design consideration for VR interventions targeting non-technical user populations. Because these findings are localized to a small deployment cohort and were not generated through a controlled effectiveness evaluation, larger controlled studies with longer-term follow-up are needed to examine program effectiveness, sustained caregiver outcomes, PLwD-related outcomes, and implementation feasibility.
